# Surgical treatment for infectious endocarditis in China

**DOI:** 10.1097/MD.0000000000041882

**Published:** 2025-03-14

**Authors:** Jing-Bin Huang, Chang-Chao Lu, Zhao-Ke Wen

**Affiliations:** a Department of Cardiothoracic Surgery, The People’s Hospital of Guangxi Zhuang Autonomous Region, Guangxi Academy of Medical Sciences, Nanning, Guangxi, China.

**Keywords:** developing countries, endocarditis, epidemiology, hospital mortality, surgery

## Abstract

This important topic of infectious endocarditis (IE) has been covered previously with large multicenter studies and reviews of national databases, most of which come from developed countries. While studies on IE in developing countries such as China are rare, a study of IE undergoing cardiac surgery in China was conducted to investigate retrospective risk factors for hospital mortality of cardiac surgery for IE. This study of patients with IE receiving cardiac surgery in the research period at our medical center was performed retrospectively; 896 patients were assigned to the hospital mortality group (n = 48) and none hospital mortality group (n = 848). Forty-eight operative deaths (5.4%) occurred. Binary logistic regression analysis for independent risk factors for hospital mortality indicated that neurological complications preoperative, destructive annulus, preoperative mitral insufficiency, postoperative left ventricular ejection fractions, and paravalvular leak are related to hospital mortality (all *P* < .001). We identified modifiable risk factors for hospital mortality of cardiac surgery for IE. Early and timely diagnosis and surgery, advancement of surgical techniques, and excellent cardiac protection may decrease hospital mortality for IE.

## 1. Introduction

Infectious endocarditis (IE) is defined as a cardiac infectious lesion, being a concerning and life-threatening disease. Despite advancements in diagnosis and treatment, the incidence rate and hospital mortality of IE are still rising. The hospital mortality rate of cardiac surgery for IE remains the highest among all valve diseases even in great heart centers. This important topic of IE has previously been covered by large multicenter studies and national database reviews, with the majority coming from developed countries.^[1–3]^ While studies on IE in developing countries are rare, there are differences in the epidemiology of IE between developed countries and developing countries.^[4]^ A study of IE undergoing cardiac surgery in China was conducted to investigate retrospective risk factors for hospital mortality of cardiac surgery for IE.

## 2. Materials and methods

### 2.1. Research design

The research of patients diagnosed with IE in our hospital from January 2006 to November 2022 was conducted retrospectively. All patient medical records were reviewed by us.

### 2.2. Diagnosis

We diagnosed the patients in the study based on modified Duke criteria,^[5]^ and surgical and pathological outcomes were reviewed to verify diagnosis before surgery.

### 2.3. Criteria of eligibility

Criteria of eligibility comprised patients diagnosed as IE during the investigation period at our medical center.

### 2.4. Technique of operation

The cardiac surgery for IE is performed based on the IE management guidelines of American Association for Thoracic Surgery. After extensive rinsing, all infected tissues and foreign objects will be thoroughly removed. We prefer allogeneic grafts for aortic root reconstruction in patients with destructive valve rings. We also preferred mitral valve repair, and replacements are performed with chordal sparing at best. Cardiac operations comprised single mitral valve operation, single aortic valve replacement operation, single tricuspid valve repair surgery, double-valve surgery, and Bentall operation plus mitral valve replacement surgery.

### 2.5. Perioperative management

After cardiac operations, all of the patients were moved to the intensive care unit (ICU). Routine anticoagulant therapy was performed. We performed transthoracic echocardiography in the ICU between 1 and 7 days after surgery.

### 2.6. Follow-up

All patients from our hospital were followed up until the end of the investigation or death. All patients underwent electrocardiogram, chest X-ray, and echocardiography examinations at our medical center, every 3 to 12 months. We linked the patients from our hospital by telephone or WeChat or interviewed them directly at our medical center at the latest follow-up.

### 2.7. Variates being analyzed

Variates were investigated (Supplementary Data, Supplemental Digital Content, http://links.lww.com/MD/O527).

The duration between symptoms and admission means the duration between the symptom beginning and the date of admission.

The duration between symptoms and surgery means the duration between the symptom beginning and the surgery date.

The hospital mortality rate means death occurs <30 days after surgery or during the same hospitalization period.

Based on the classification of Kidney Disease Improving Global Outcomes, if creatinine of serum elevates by ≥26.5 μmol/L (0.3 mg/dL) within 48 hours, creatinine of serum is half greater than the baseline within the first 7 days, or urine volume is <0.5 mL/kg/hour for 6 hours, the patient is diagnosed as acute kidney injury.^[6]^

Multiorgan failure (MOF) is considered to be a continuous process of all kinds of levels of organ failure instead of an all-or-none event, with 6 “key organs”: central nervous system, cardiovascular system, lungs, liver, kidneys, and coagulation system.^[7]^

### 2.8. Statistics

We reported continuous variables as mean ± SE and performed the Kolmogorov-Smirnov test on the normal distribution of all variables in the research.

Survival rates of patients in the study were accessed by the method of Kaplan-Meier. We used the χ^2^ test, the Kruskal-Walls test, or the Wilcoxon rank-sum test to investigate relationships between the preoperative, intraoperative, and postoperative variates. The relationships of risk factors perioperative were investigated by contingency table methods and binary logistic regression analysis. *P* values of <0.05 were considered to be statistically significant. We have done all analyses using IBM SPSS version 24.0 software (IBM SPSS Inc, USA).

## 3. Outcomes

### 3.1. Base characteristics of the patients

Two thousand sixteen patients diagnosed as IE were enrolled in the research: 896 (44.4%, 896/2016) underwent surgery, 224 (11.1%, 224/2016) received medical therapy alone due to absence of surgical indication, 432 (21.4%, 432/2016) did not undergo cardiac operation due to MOF at admission, and 464 (23.0%, 464/2016) did not undergo cardiac operation, although with surgical indication, due to refusal of patients and their relatives.

### 3.2. Outcomes of operations

Eight hundred ninety-six patients with IE undergoing cardiac surgery were further assigned to the hospital mortality group (n = 48) and none hospital mortality group (n = 848). Forty-eight operative deaths (48/896, 5.4%) occurred (Tables 1 and 2).

**Table 1:** Characteristics of the operative patients (n = 896).

Variable	Value
Female/male, n	304/592
Age, yr	38.45 ± 0.48 (8–71)
Weight, kg	55.48 ± 0.40 (19–89)
Body mass index, kg/m^2^	21.8 ± 0.4 (15.6–26.1)
Time between symptoms and surgery, mo	2.46 ± 0.07 (0.1–12)
NYHA class	
II, n	513 (57.3%)
III, n	251 (28.0%)
IV, n	132 (14.7%)
Comorbidities	
Congenital heart disease, n	32 (3.6%)
Coronary heart disease, n	18 (2.0%)
Hypertension, n	36 (4%)
Diabetes mellitus, n	17 (1.9%)
Preoperative left ventricular end diastolic dimension, mm	61.07 ± 0.31 (36–84)
Preoperative left ventricular ejection fractions, %	62.0 ± 0.3 (39–74)
Preoperative aortic insufficiency, cm^2^	5.51 ± 0.23 (0–28)
Preoperative mitral insufficiency, cm^2^	7.27 ± 0.21 (0–19.5)
Preoperative tricuspid insufficiency, cm^2^	4.67 ± 0.16 (0–16.9)
Vegetation length, mm	10.19 ± 0.23 (2–28)
Neurological complications before surgery, n	112 (13.6%)
Native valve IE	800 (89.3%)
Prosthetic valve IE	96 (10.7%)
Microbiology	
Negative blood culture	567 (63.3%)
*Staphylococcus aureus* endocarditis	98 (10.9%)
Streptococci endocarditis	161 (18.0%)
Other	70 (13.6%)
Serum creatinine before surgery, μmol/L	81.18 ± 1.12 (79–241)
Operation	
Isolated aortic valve replacement, n	176 (13.6%)
Isolated mitral valve surgery, n	368 (41.1%)
Double-valve operation, n	256 (28.6%)
Isolated tricuspid annuloplasty, n	80 (8.92%)
Bentall + mitral valve replacement, n	16 (1.8%)
Extracorporeal membrane oxygenation, n	3 (0.3%)

IE = infectious endocarditis, NYHA = New York Heart Association.

**Table 2:** Preoperative data (n = 896).

Variable	Hospital mortality group (n = 48)	Nonhospital mortality group (n = 848)	*P* value
Male, n (%)	38 (70.8%)	592 (69.8%)	<.001
Age, yr	43.63 ± 0.72	41.45 ± 0.50	.955
Weight, kg	52.33 ± 1.11	55.66 ± 0.41	.059
Duration between symptoms and surgery, mo	4.27 ± 0.43	2.36 ± 0.07	<.001
Vegetation length, mm	15.67 ± 0.48	9.88 ± 0.23	<.001
Preoperative left ventricular end diastolic dimension, mm	62.0 ± 1.10	61.02 ± 0.33	.477
Preoperative left ventricular ejection fractions, %	69.0 ± 1.00	62.0 ± 0.30	<.001
Preoperative aortic insufficiency, cm^2^	7.00 ± 0.72	5.43 ± 0.24	.119
Preoperative mitral insufficiency, cm^2^	5.33 ± 0.22	7.38 ± 0.22	.027
Preoperative tricuspid insufficiency, cm^2^	4.00 ± 0.41	4.71 ± 0.17	.315
Neurological complications before surgery, n	16 (33.3%)	96 (11.3%)	<.001
Serum creatinine before surgery, μmol/L	160.3 ± 10.37	76.70 ± 0.79	<.001

The duration between symptoms and surgery (4.27 ± 0.43 vs 2.36 ± 0.07 months), left ventricular end diastolic dimension preoperative (69.0 ± 1.0% vs 62.0 ± 0.3%), vegetation length (15.67 ± 0.48 vs 9.88 ± 0.23 mm), and creatinine of serum preoperative (160.3 ± 10.37 vs 76.70 ± 0.79 μmol/L) in the hospital mortality group were markedly higher than those in none hospital mortality group (all *P* < .001; Tables 1 and 2).

### 3.3. Resource utilization

Duration of aortic cross-clamp (115.7 ± 6.0 vs 84.8 ± 1.2 minutes), cardiopulmonary bypass duration (175.7 ± 7.8 vs 138.6 ± 1.8 minutes), mechanical ventilation duration (219.7 ± 11.7 vs 38.4 ± 1.4 hours), ICU retention time (9.7 ± 0.5 vs 4.5 ± 0.1 days), creatinine of serum 24 hours after surgery (151.7 ± 12.2 vs 84.9 ± 1.3 μmol/L), creatinine of serum 48 hours after surgery (217.7 ± 12.5 vs 94.7 ± 1.9 μmol/L), thoracic drainage (753.3 ± 68.1 vs 617.7 ± 13.5 mL; *P* = .022), left ventricular end diastolic dimension postoperative (53.3 ± 0.25 vs 47.63 ± 0.25 mm), fresh-frozen plasma transfusion (980.0 ± 60.8 vs 601.1 ± 15.9 mL), and packed red cells’ transfusion (10.0 ± 0.9 vs 2.3 ± 0.1 units) in the hospital mortality group were markedly higher than those in none hospital mortality group (all *P* < .001 except *P* of thoracic drainage; Table 3).

**Table 3:** Operative data (n = 896).

Variable	Hospital mortality group (n = 48)	Nonhospital mortality group (n = 848)	*P* value
Aortic cross-clamp duration, min	115.7 ± 6.0	84.8 ± 1.2	<.001
Cardiopulmonary bypass duration, min	175.7 ± 7.8	138.6 ± 1.8	<.001
Mechanical ventilation time, h	219.7 ± 11.7	38.4 ± 1.4	<.001
ICU retention time, d	9.7 ± 0.5	4.5 ± 0.1	<.001
Hospitalized time postoperative, d	9.7 ± 0.5	19.5 ± 0.3	<.001
Serum creatinine 24 hours after surgery, μmol/L	151.7 ± 12.2	84.9 ± 1.3	<.001
Serum creatinine 48 hours after surgery, μmol/L	217.7 ± 12.5	94.7 ± 1.9	<.001
Fluid balance on operation day, mL	−233.3 ± 84.5	−634.2 ± 27.0	.001
Fluid balance on first day post-operative, mL	416.7 ± 113.7	−674.5 ± 36.7	<.001
Fluid balance on second day post-operative, mL	−33.3 ± 49.6	−575.5 ± 25.1	<.001
Chest drainage, mL	753.3 ± 68.1	617.7 ± 13.5	.022
Postoperative left ventricular end diastolic dimension, mm	53.3 ± 2.5	47.63 ± 1.5	<.001
Postoperative left ventricular ejection fractions, %	57.0 ± 0.1	59.0 ± 0.2	.045
Dose of adrenaline, μg/kg/min	1.51 ± 0.02	0.04 ± 0.01	<.001
Blood lactate, mmol/L	12.6 ± 0.7	2.2 ± 0.1	<.001
Fresh-frozen plasma, mL	980.0 ± 60.8	601.1 ± 15.9	<.001
Packed red cells, units	10.0 ± 0.9	2.3 ± 0.1	<.001

ICU = intensive care unit.

Fluid balance on operation day (−233.3 ± 84.5 vs −634.2 ± 27.0 mL; *P* = .001), fluid balance on first day postoperative (416.7 ± 113.7 vs −674.5 ± 36.7 mL; *P* < .001), and fluid balance on second day postoperative (−33.3 ± 49.6 vs −575.5 ± 25.1 mL; *P* < .001) in the hospital mortality group were markedly less negative than those in none hospital mortality group (Table 3).

Postoperative left ventricular ejection fractions (LVEFs; 57.0 ± 0.1 vs 59.0 ± 0.2%; *P* = .045) in the hospital mortality group were markedly less than that in none hospital mortality group (Table 3).

The common early postoperative complications included postoperative acute renal injury (30.4%, 272/896), long-term mechanical ventilation duration >24 hours (39.3%, 352/896), and MOF (9.6%, 86/896; Table 4).

**Table 4:** Postoperative mortality and complications (n = 896).

Causes of postoperative mortality	n (%)
Paravalvular leak + cardiogenic shock + acute renal injury + hepatic failure + septicemia	32 (3.6%)
Intracerebral hemorrhage	16 (1.8%)
Complications	
Acute renal injury, n	272 (30.4%)
Mechanical ventilation time >24 h	352 (39.3%)
Hepatic failure, n	39 (4.4%)
Respiratory failure, n	142 (15.8%)
Ventricular fibrillation, n	33 (3.7%)

### 3.4. Risk factors for hospital mortality after cardiac surgery

Binary logistic regression analysis of potential risk factors for hospital mortality indicated that factors are related to hospital mortality, such as neurological complications before surgery (odds ratio [OR], 9.910 [95% CI, 4.337–22.43]), destructive annulus (OR, 4.165 [95% CI, 2.327–8.176]), mitral insufficiency preoperative (OR, 1.290 [95% CI, 1.144–1.456]), postoperative LVEFs (OR, 3.243 [95% CI, 1.081–7.56]), and paravalvular leak (OR, 8.478 [95% CI, 5.95–14.96]; all *P* < .001; Table 5).

**Table 5:** Analysis of risk factors for hospital mortality following cardiac surgery.

Model	OR	95% CI	*P* value
Univariate analysis of risk factors for hospital mortality			
Neurological complications before surgery	3.917	2.072–7.403	<.001
Destruction of the annulus	6.125	3.191–11.756	<.001
Preoperative mitral regurgitation	1.058	1.006–1.114	.029
Postoperative left ventricular ejection fractions	5.243	1.081–8.56	.046
Paravalvular leak	9.333	6.107–14.950	<.001
Multivariate analysis of risk factors for hospital mortality			
Neurological complications before surgery	9.910	4.337–22.43	<.001
Destruction of the annulus	4.165	2.327–8.176	<.001
Preoperative mitral regurgitation	1.290	1.144–1.456	<.001
Postoperative left ventricular ejection fractions	3.243	1.081–7.56	<.001
Paravalvular leak	8.478	5.95–14.96	<.001

OR = odds ratio.

### 3.5. Outcomes of follow-up

**Figure 1.**
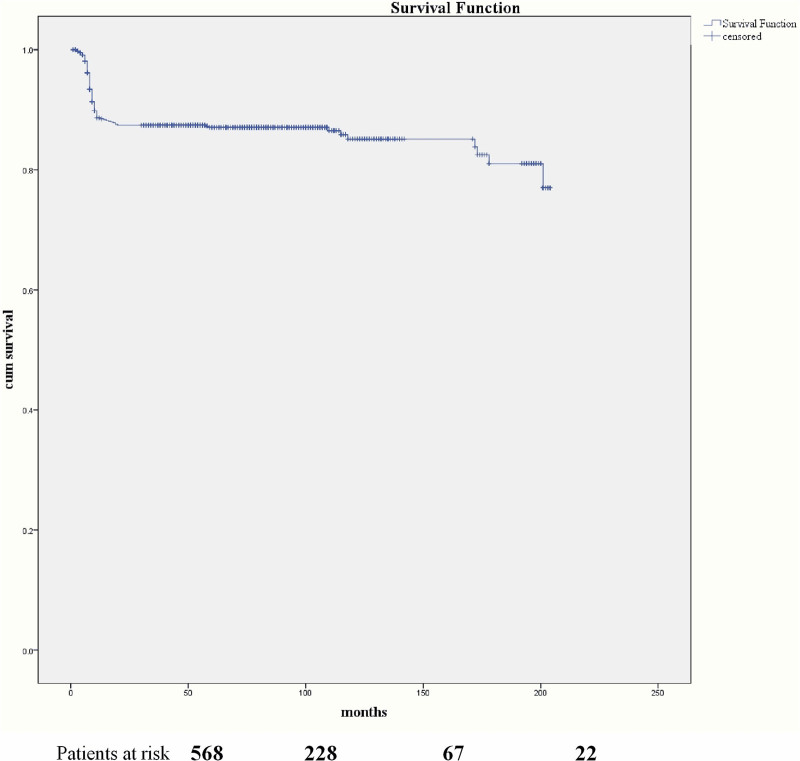
Kaplan-Meier curve for survival. The follow-up of 814 patients was completed (96.0%, 814/848). Eighty-seven deaths (10.7%, 87/814) occurred within 12 months after being discharged from our hospital because of recurrence of infectious endocarditis (IE) and cerebral hemorrhage.

Survivors (n = 848) were discharged from our medical center, and 814 patients were followed up to the end date of this research or death date; 96.0% (814/848) was completed in follow-up. The average follow-up length was 75.14 ± 1.80 (1–204) months. Eighty-seven deaths (87/814, 10.7%) happened in 1 year during follow-up due to IE recurrence and cerebral hemorrhage. The last follow-up demonstrated 681 patients discharged from our medical center in New York Heart Association class I (681/707, 96.3%) and 26 in class II (26/707, 3.7%; Fig. 1).

## 4. Discussion

IE is a complex and progressive disease, with great mortality (about 20%–25%), high incidence rate, long hospitalization stay, and a heavy burden on the medical system. The incidence rate of IE remains elevated all over the world despite the improvements in diagnosis and treatment.^[8–11]^ Apparent changes have happened recently in the epidemiology of IE in the world. The average age of patients with healthcare-related, artificial valve–related, and device-related IE, infection of coagulase-negative staphylococcal, and associated complications has all elevated. However, the prognosis of IE has become better recently with an apparent decrease in hospital and 6-month mortality rates although the complexity of cases of IE elevates.^[12,13]^

A study of patients with IE undergoing cardiac surgery in China was conducted to investigate retrospective risk factors for hospital mortality from heart surgery. In our study, we identified preoperative neurological complications, destructive annulus, preoperative mitral insufficiency, postoperative LVEFs, and paravalvular leak to be risk factors for hospital mortality of cardiac surgery for IE.

Currently, in high-income countries, 50% to 60% of patients undergo IE surgery, with a 6-month survival rate of >80%.^[14–16]^ In the present investigation, we diagnosed 2016 patients as IE in the period of investigation, and 44.4% (896/2016) underwent cardiac surgery. There was a distinction of time point between the hospital mortality group and none hospital mortality group (≈4+ vs 2+ months). Because of the absence of excellent primary, secondary, and tertiary prevention networks, the patients with IE in our study saw a doctor late and were commonly diagnosed later and transferred to our tertiary medical center. Therefore, timely and early diagnosis and intervention of IE are vital. Advocate early surgical management for IE is progressive and life-threatening, and patients with poor preoperative cardiac function are graded with the greatest hospital mortality rate. The quick and accurate diagnosis of patients with IE is still the grave challenge of this disease. Social and economic factors affect the characteristics in clinics of patients with IE worldwide. Although being younger, the patients in the developing countries experience a higher incidence rate of complications and greater mortality rates, which are related to delayed diagnosis and less surgical intervention. Delayed diagnosis and intervention can result in more complications and worse clinical results.^[17]^

The American Association for Thoracic Surgery guideline emphasizes that once the surgical indications are determined, cardiac surgery should be conducted as soon as possible. A system of primary, secondary, and tertiary prevention consisting of needed basic information and necessary conditions should be established for IE control.^[18–22]^ For the complications of IE including thrombotic events, congestive heart failure, and valve abscess, early surgery is recommended. In developing countries, the complications above are more common in most patients with IE, as the patients are typically diagnosed later and hospitalized later. In order to improve the effectiveness of IE in developing countries, the scope of cardiac surgery interventions should be expanded.^[23–25]^

Destructive annulus and preoperative mitral insufficiency indicate the severity of the disease. Patients with annulus destruction are prone to early perivalvular leakage, which is a grave complication that apparently increases hospital mortality. Early and timely diagnosis and treatment will stop or prevent the progress of periannular abscess and insults to the valve annulus. Surgical technique improvement will decrease the incidence rate of perivalvular leakage. However, perivalvular leakage and annulus damage in patients with IE remain a challenging issue for cardiac surgeons.

Increasing evidence suggests that aortic root replacement operation is related to an excellent incidence rate of perivalvular leakage and reoperation, a hypothesis intuitive or supported by established operation principles. Patients undergoing aortic root replacement operation had less risk of reoperation and incidence rate of early perivalvular leakage in 1-year follow-up. Some researchers suggest that aortic root replacement surgery is the best practice choice for treating aortic valve endocarditis with peritubular abscess and aortic ring destruction. Destructive aortic valve annulus is associated with early aortic valve leakage, hospital mortality, and 1-year mortality. Early perivalvular leakage and sepsis are the main causes of hospital mortality. Early perivalvular leakage mainly occurs in patients with destructive aortic valve rings, which are sutured with 6.0-Polene and reconstructed with patches. Aortic valve leakage usually does not occur after Bentall surgery. Postoperative LVEF and perivalvular leakage are risk factors for hospital mortality in patients with infective endocarditis undergoing cardiac surgery. Good heart protection is of great significance for hospital mortality.^[26–31]^

In the present study of our cohort, the main reason for hospital mortality includes septic shock with consecutive MOF and cerebral hemorrhage. The mean follow-up length was 75.14 ± 1.80 months. Eighty-seven deaths (87/814, 10.7%) happened in 1 year after being discharged from our medical center because of recurrence of IE and cerebral hemorrhage. Data of follow-up on incidence rate and mortality indicate that almost all deaths occur in 1 year following surgery, meaning that all patients discharged from the hospital should have close follow-up.

Patients with preoperative neurological complications are more serious, which, to some extent, clarifies that the reasons for preoperative neurological complications are associated with greater hospital mortality rates and longer mechanical ventilation duration. An investigation showed that only 25.9% of patients with symptomatic neurological complications need operation at admission, while the remaining 74.1% of patients are contraindicated for operation because of death, severe septic shock, stroke, and coma, or extensive neurological deficits.^[32–35]^ The prognosis of left-sided IE is determined by multiple factors, including vegetation size. The cutoff value for vegetation length used to predict embolic events or 30-day mortality is >10 mm.^[36]^ Delayed operation for patients with IE following ischemic stroke does not bring apparent benefits for survival. Further observation and analysis such as detailed preoperative and postoperative clinical neurological findings and advanced imaging data including the size of ischemic stroke can provide more accurate information for the optimal timing of valve operation in patients with IE and recent stroke syndrome.^[37]^

Effective and timely diagnosis and management of neurological complications remain huge challenges we face. The contribution of repeated transthoracic or transesophageal echocardiography to the diagnosis of IE reduces with increasing repetition times. No evidence showed that >3× repeated examinations of transthoracic or transesophageal echocardiography were an effective strategy to improve the diagnostic rate of all endocarditis patients except for some selected suspected patients.^[38,39]^ In our investigation, twice transthoracic echocardiographies by different ultrasound doctors were performed to the diagnosis of endocarditis. Effective diagnosis and treatment of neurological complications are still grave challenges we face. The contribution of repeated echocardiography (transthoracic or transesophageal) to the diagnosis of endocarditis reduces with elevating repetition times. In our study, different ultrasound doctors performed 2 transthoracic echocardiography examinations to diagnose endocarditis.

### 4.1. Perspectives

How to provide optimal treatment for patients with IE is still a daunting challenge we face. A network of primary, secondary, and tertiary prevention and early and timely diagnosis and intervention of IE will help achieve better short- and long-term results.

### 4.2. Advantages and limitations of research

In the study completed by Huang et al,^[11]^ the factors associated with the prolongation of ICU stay after cardiac surgery for IE were identified. Therefore, the shortening of ICU stay and the optimization of preoperative, intraoperative, and postoperative factors to shorten ICU stay will help to achieve better postoperative results and reduce mortality and incidence rates.^[11]^ A study on the surgical treatment of left IE with symptomatic neurological complications preoperative in China has shown that symptomatic neurological complications before surgery are related to greater hospital mortality and prolonged mechanical ventilation duration following cardiac surgery.^[28]^ We also reported the modifiable risk factors of immediate and long-term outcomes in the operable and inoperable with left-sided infective endocarditis.^[40]^ In the present study, we focused on the left- and right-sided infective endocarditis and investigated risk factors for hospital mortality of cardiac surgery for IE and identified neurological complications before surgery, destructive annulus, preoperative mitral insufficiency, postoperative LVEFs, and paravalvular leak to be associated with hospital mortality, which adds some value to the current literature.

The shortcomings of the research lie in its single-center retrospective maturity. Because of the retrospective nature of the research and the functions of our medical center as a referral hospital, selection bias will exist. Well-designed studies, such as prospective multiple-center cohort studies, are advocated, and projects to reduce the incidence rate and mortality of IE inpatients in developing countries are needed.

## 5. Conclusion

We identified modifiable risk factors for hospital mortality of cardiac surgery for IE. Early and timely diagnosis and surgery, improvement of surgical techniques, and excellent cardiac protection may decrease hospital mortality for IE.

## Acknowledgments

The authors thank Drs Li and Long for their help.

## Author contributions

**Conceptualization:** Jing-Bin Huang.

**Formal analysis:** Jing-Bin Huang.

**Funding acquisition:** Jing-Bin Huang.

**Writing – original draft:** Jing-Bin Huang.

**Resources:** Chang-Chao Lu.

**Validation:** Chang-Chao Lu.

**Writing – review & editing:** Chang-Chao Lu, Zhao-Ke Wen.

**Methodology:** Zhao-Ke Wen.

**Software:** Zhao-Ke Wen.

**Visualization:** Zhao-Ke Wen.

## Supplementary Material


